# Supplementary material for: Phylogenetic comparison between Type IX Secretion System (T9SS) protein components suggests evidence of horizontal gene transfer
Source: PeerJ. 2020 Jun 26;8:e9019. doi: 10.7717/peerj.9019 (PMC7323717; doi:10.7717/peerj.9019)
Supplement: Supplemental Information 1 — T9SS components sequences, alignment of each T9SS protein family, NEX files used as input for MrBayes tool, genome annotation (GFF) file for Porphyromonas gingivalis and detailed Bayesian Inference trees with support values. [file peerj-08-9019-s001.zip › supplemental/Supplemental files/Supplemental Table S1.docx]

**Table S1:**

**The species that are represented by terminal nodes of 19 BI trees that are out of its expected monophyletic clades.**

| **BI trees** | **Classes** | **Species out of its expected monophyletic clade** |
| --- | --- | --- |
| Omp17 | Flavobacteriia | *Owenweeksia hongkongensis* DSM 17368 |
|  |  | *Fluviicola taffensis* DSM 16823 |
|  |  | *Elizabethkingia miricola* |
|  |  | *Elizabethkingia anophelis* FMS-007 |
|  |  | *Elizabethkingia anophelis* NUHP1 |
|  |  | *Elizabethkingia anophelis* |
|  |  | *Capnocytophaga ochracea* DSM 7271 |
|  |  | *Capnocytophaga sp.* oral taxon 323 |
|  |  | *Capnocytophaga haemolytica* |
|  | Cytophagia | *Cardinium endosymbiont cEper1 of Encarsia pergandiella* |
|  |  | *Hymenobacter sp.* PAMC 26554 |
|  |  | *Candidatus Amoebophilus asiaticus* 5a2 |
|  | Bacteroidia | *Candidatus Azobacteroides pseudotrichonymphae* genomovar. CFP2 |
|  |  | *Alistipes finegoldii* DSM 17242 |
|  |  | Bacteroidales bacterium CF |
|  |  | bacterium L21-Spi-D4 |
|  |  | *Odoribacter splanchnicus* DSM 20712 |
|  |  | *Draconibacterium orientale* |
| PorE | Flavobacteriia | *Owenweeksia hongkongensis* DSM 17368 |
|  |  | *Fluviicola taffensis* DSM 16823 |
|  | Cytophagia | *Cytophaga hutchinsonii* ATCC 33406 |
|  |  | *Hymenobacter sp.* DG25B |
|  |  | *Hymenobacter sp.* PAMC 26554 |
|  |  | *Candidatus Amoebophilus asiaticus* 5a2 |
|  |  | *Bernardetia litoralis* DSM 6794 |
|  |  | *Pontibacter korlensis* |
| PorF | Flavobacteriia | *Fluviicola taffensis* DSM 16823 |
|  | Bacteroidia | bacterium L21-Spi-D4 |
|  |  | *Alistipes finegoldii* DSM 17242 |
|  |  | *Bacteroides salanitronis* DSM 18170 |
| PorG | Bacteroidia | bacterium L21-Spi-D4 |
|  | Cytophagia | *Runella slithyformis* DSM 19594 |
|  | Flavobacteriia | *Fluviicola taffensis* DSM 16823 |
| PorK | Bacteroidia | *Odoribacter splanchnicus* DSM 20712 |
| PorL | Flavobacteriia | *Fluviicola taffensis* DSM 16823 |
| PorM | Flavobacteriia | *Owenweeksia hongkongensis* DSM 17368 |
|  |  | *Fluviicola taffensis* DSM 16823 |
| PorN | Bacteroidia | *Draconibacterium orientale* |
|  |  | bacterium L21-Spi-D4 |
| PorP | Flavobacteriia | *Psychroflexus torquis* ATCC 700755 |
|  |  | *Owenweeksia hongkongensis* DSM 17368 |
|  |  | Flavobacteriaceae bacterium UJ101 |
|  |  | *Ornithobacterium rhinotracheale* DSM 15997 |
|  |  | *Ornithobacterium rhinotracheale* ORT-UMN 88 |
|  | Bacteroidia | *Draconibacterium orientale* |
|  |  | bacterium L21-Spi-D4 |
|  | Cytophagia | *Rufibacter sp.* DG15C |
|  |  | *Rufibacter sp.* DG31D |
|  |  | *Rufibacter tibetensis* |
|  |  | *Pontibacter korlensis* |
|  |  | *Bernardetia litoralis* DSM 6794 |
|  |  | *Marivirga tractuosa* DSM 4126 |
|  |  | *Flammeovirga sp.* MY04 |
|  |  | *Runella slithyformis* DSM 19594 |
|  |  | *Leadbetterella byssophila* DSM 17132 |
|  |  | *Dyadobacter fermentans* DSM 18053 |
|  |  | *Cyclobacterium amurskyense* |
| PorQ | Flavobacteriia | *Fluviicola taffensis* DSM 16823 |
|  | Bacteroidia | bacterium L21-Spi-D4 |
| PorT | Flavobacteriia | *Fluviicola taffensis* DSM 16823 |
| PorU | Cytophagia | *Hymenobacter sp.* DG5B |
|  |  | *Hymenobacter sp.* PAMC 26628 |
|  |  | *Hymenobacter sp.* PAMC 26554 |
|  |  | *Hymenobacter sp.* APR13 |
|  |  | *Hymenobacter swuensis* DY53 |
|  |  | *Hymenobacter sp.* DG25B |
|  |  | *Hymenobacter sp.* DG25A |
| PorV | Flavobacteriia | *Owenweeksia hongkongensis* DSM 17368 |
|  |  | *Fluviicola taffensis* DSM 16823 |
|  | Bacteroidia | *Draconibacterium orientale* |
|  |  | bacterium L21-Spi-D4 |
|  |  | *Odoribacter splanchnicus* DSM 20712 |
|  |  | *Bacteroides caecimuris* |
|  |  | *Bacteroides ovatus* |
|  |  | *Bacteroides ovatus* V975 |
|  |  | *Alistipes finegoldii* DSM 17242 |
|  |  | *Bacteroides dorei* |
|  |  | *Bacteroides dorei* CL03T12C01 |
| PorW | Flavobacteriia | *Fluviicola taffensis* DSM 16823 |
|  | Bacteroidia | bacterium L21-Spi-D4 |
|  |  | *Draconibacterium orientale* |
| PorX | Flavobacteriia | *Fluviicola taffensis* DSM 16823 |
|  | Cytophagia | *Cytophaga hutchinsonii* ATCC 33406 |
| PorY | Flavobacteriia | *Owenweeksia hongkongensis* DSM 17368 |
|  |  | *Fluviicola taffensis* DSM 16823 |
|  |  | *Ornithobacterium rhinotracheale* ORT-UMN 88 |
|  |  | *Ornithobacterium rhinotracheale* DSM 15997 |
|  |  | Flavobacteriaceae bacterium UJ101 |
|  |  | *Weeksella virosa* DSM 16922 |
|  |  | *Riemerella anatipestifer* RA-CH-1 |
|  |  | *Riemerella anatipestifer* CH3 |
|  |  | *Riemerella anatipestifer* ATCC 11845 DSM 15868 |
|  |  | *Riemerella anatipestifer* |
|  |  | *Riemerella anatipestifer* RA-CH-2 |
|  |  | *Riemerella anatipestifer* Yb2 |
|  |  | *Elizabethkingia miricola* |
|  |  | *Elizabethkingia anophelis* NUHP1 |
|  |  | *Elizabethkingia anophelis* |
|  |  | *Elizabethkingia anophelis* FMS-007 |
|  |  | Flavobacteriaceae bacterium 3519-10 |
|  |  | *Chryseobacterium sp.* IHB B 17019 |
|  |  | *Chryseobacterium sp.* IHB B 10212 |
|  |  | *Chryseobacterium gallinarum* |
|  |  | *Chryseobacterium sp.* StRB126 |
|  | Bacteroidia | *Alistipes finegoldii* DSM 17242 |
|  |  | *Mucinivorans hirudinis* |
|  |  | *Tannerella forsythia* 3313 |
|  |  | *Draconibacterium orientale* |
| PorZ | Flavobacteriia | *Fluviicola taffensis* DSM 16823 |
|  | Bacteroidia | bacterium L21-Spi-D4 |
| SigP | Flavobacteriia | *Capnocytophaga haemolytica* |
|  |  | *Ornithobacterium rhinotracheale* DSM 15997 |
|  |  | *Ornithobacterium rhinotracheale* ORT-UMN 88 |
|  |  | *Capnocytophaga sp.* oral taxon 323 |
|  |  | *Capnocytophaga canimorsus* Cc5 |
|  | Bacteroidia | *Draconibacterium orientale* |
|  |  | bacterium L21-Spi-D4 |
|  |  | *Alistipes finegoldii* DSM 17242 |
|  |  | *Mucinivorans hirudinis* |
|  |  | *Porphyromonas gingivalis* |
|  |  | *Porphyromonas gingivalis* W83 |
|  |  | *Porphyromonas gingivalis* ATCC 33277 |
|  |  | *Porphyromonas gingivalis* TDC60 |
|  |  | *Porphyromonas gingivalis* 381 |
|  |  | *Porphyromonas gingivalis* AJW4 |
|  |  | *Porphyromonas gingivalis* A7A1-28 |
|  |  | *Porphyromonas asaccharolytica* DSM 20707 |
|  |  | *Bacteroides salanitronis* DSM 18170 |
|  |  | *Bacteroides ovatus* V975 |
|  |  | *Bacteroides ovatus* |
|  |  | *Bacteroides caecimuris* |
|  |  | Bacteroidales bacterium CF |
|  |  | *Odoribacter splanchnicus* DSM 20712 |
|  |  | *Bacteroides helcogenes* P 36-108 |
|  |  | *Bacteroides dorei* CL03T12C01 |
|  |  | *Bacteroides dorei* |
| Sov | Flavobacteriia | *Fluviicola taffensis* DSM 16823 |
