# Supplementary figures and images for: Phylogenetic comparison between Type IX Secretion System (T9SS) protein components suggests evidence of horizontal gene transfer
Source: PeerJ. 2020 Jun 26;8:e9019. doi: 10.7717/peerj.9019 (PMC7323717; doi:10.7717/peerj.9019)

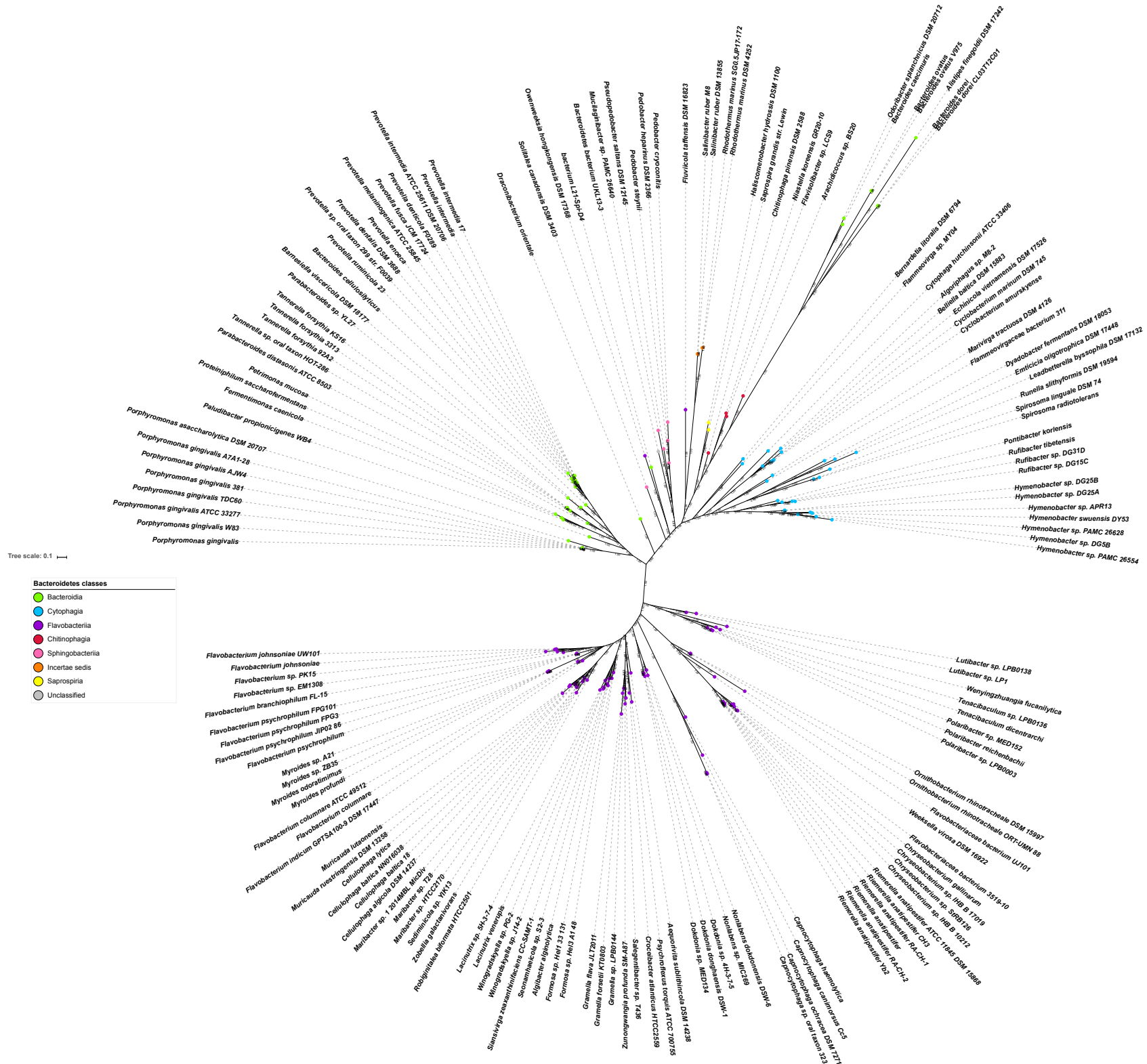

**Figure S11: The BI tree of PorV with terminal nodes labelled with its corresponding species.**

Supplement: Supplemental Information 1 — T9SS components sequences, alignment of each T9SS protein family, NEX files used as input for MrBayes tool, genome annotation (GFF) file for Porphyromonas gingivalis and detailed Bayesian Inference trees with support values. [file peerj-08-9019-s001.zip › supplemental/Supplemental files/Supplemental Figure S11.pdf]

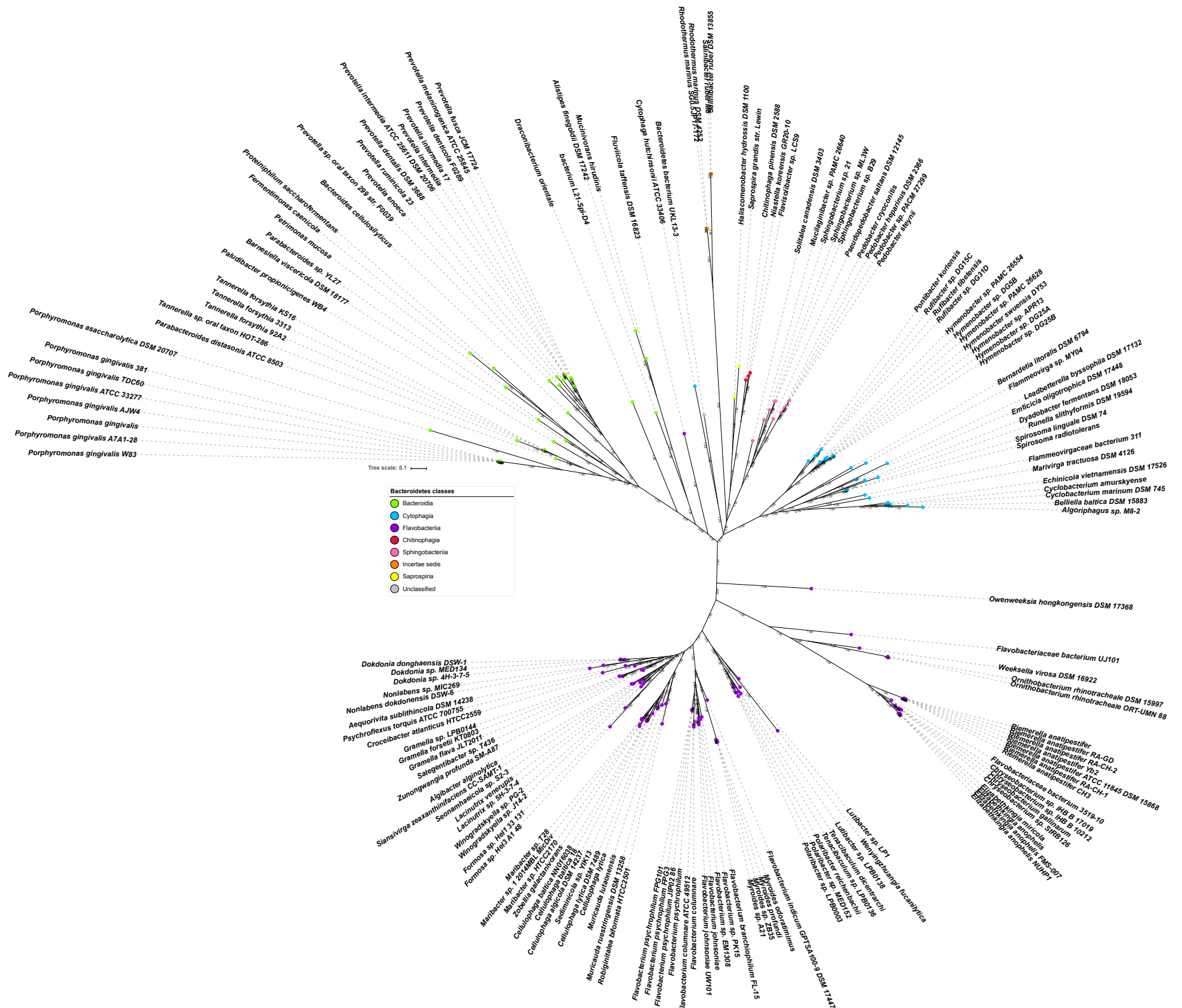

Figure S13: The BI tree of PorX with terminal nodes labelled with its corresponding species.

Supplement: Supplemental Information 1 — T9SS components sequences, alignment of each T9SS protein family, NEX files used as input for MrBayes tool, genome annotation (GFF) file for Porphyromonas gingivalis and detailed Bayesian Inference trees with support values. [file peerj-08-9019-s001.zip › supplemental/Supplemental files/Supplemental Figure S13.pdf]

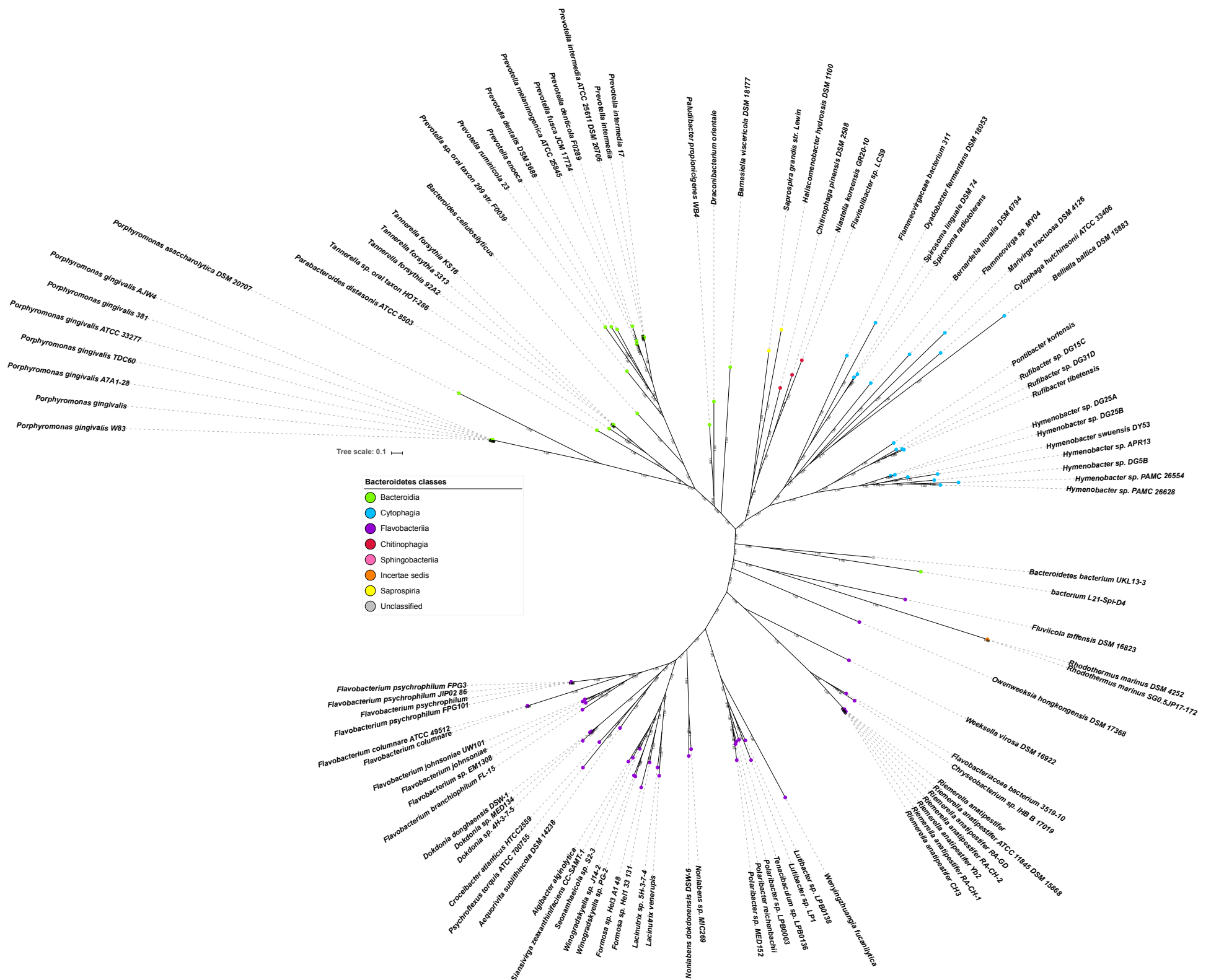

**Figure S15: The BI tree of PorZ with terminal nodes labelled with its corresponding species.**

Supplement: Supplemental Information 1 — T9SS components sequences, alignment of each T9SS protein family, NEX files used as input for MrBayes tool, genome annotation (GFF) file for Porphyromonas gingivalis and detailed Bayesian Inference trees with support values. [file peerj-08-9019-s001.zip › supplemental/Supplemental files/Supplemental Figure S15.pdf]

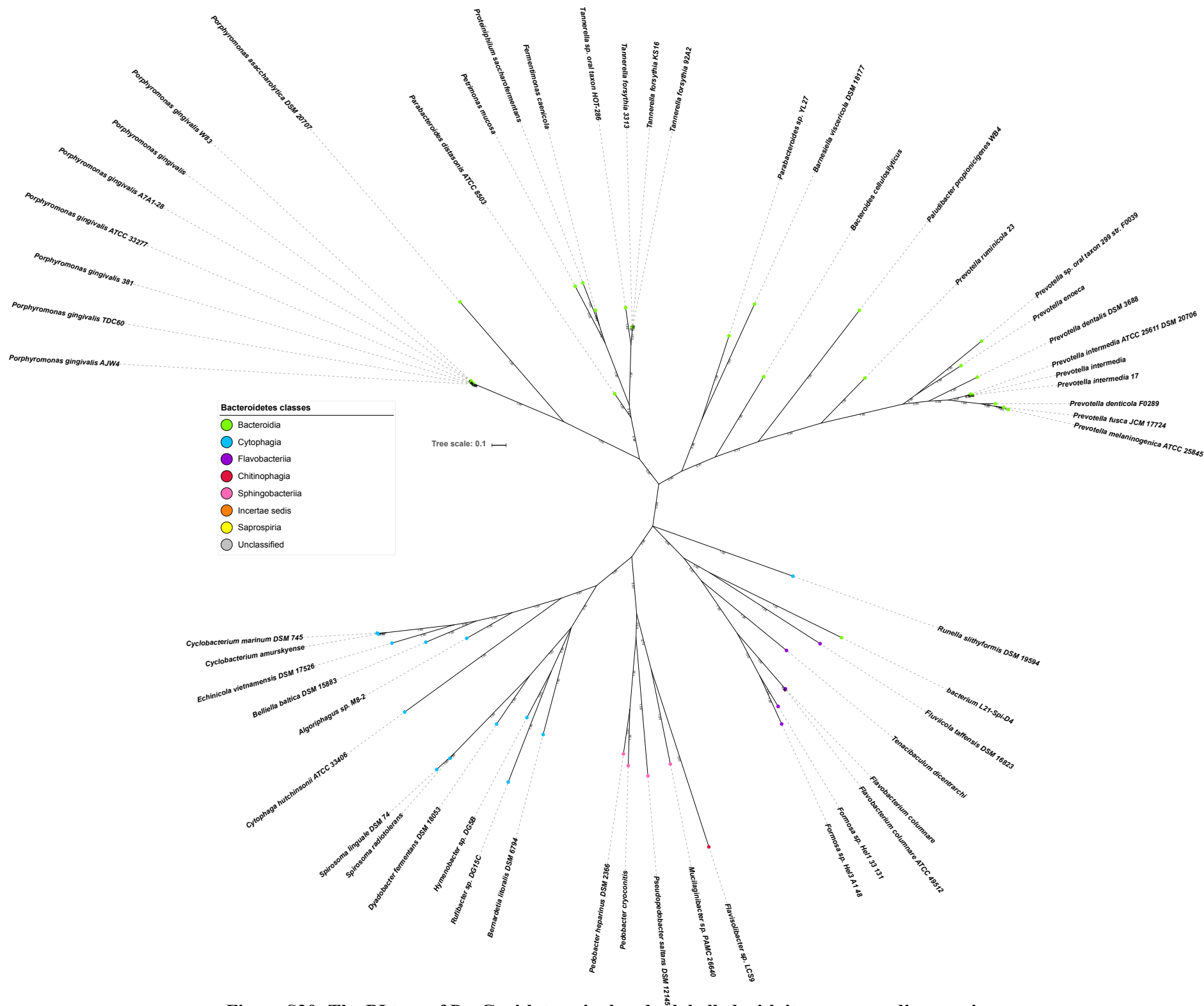

**Figure S20: The BI tree of PorG with terminal nodes labelled with its corresponding species.**

Supplement: Supplemental Information 1 — T9SS components sequences, alignment of each T9SS protein family, NEX files used as input for MrBayes tool, genome annotation (GFF) file for Porphyromonas gingivalis and detailed Bayesian Inference trees with support values. [file peerj-08-9019-s001.zip › supplemental/Supplemental files/Supplemental Figure S20.pdf]

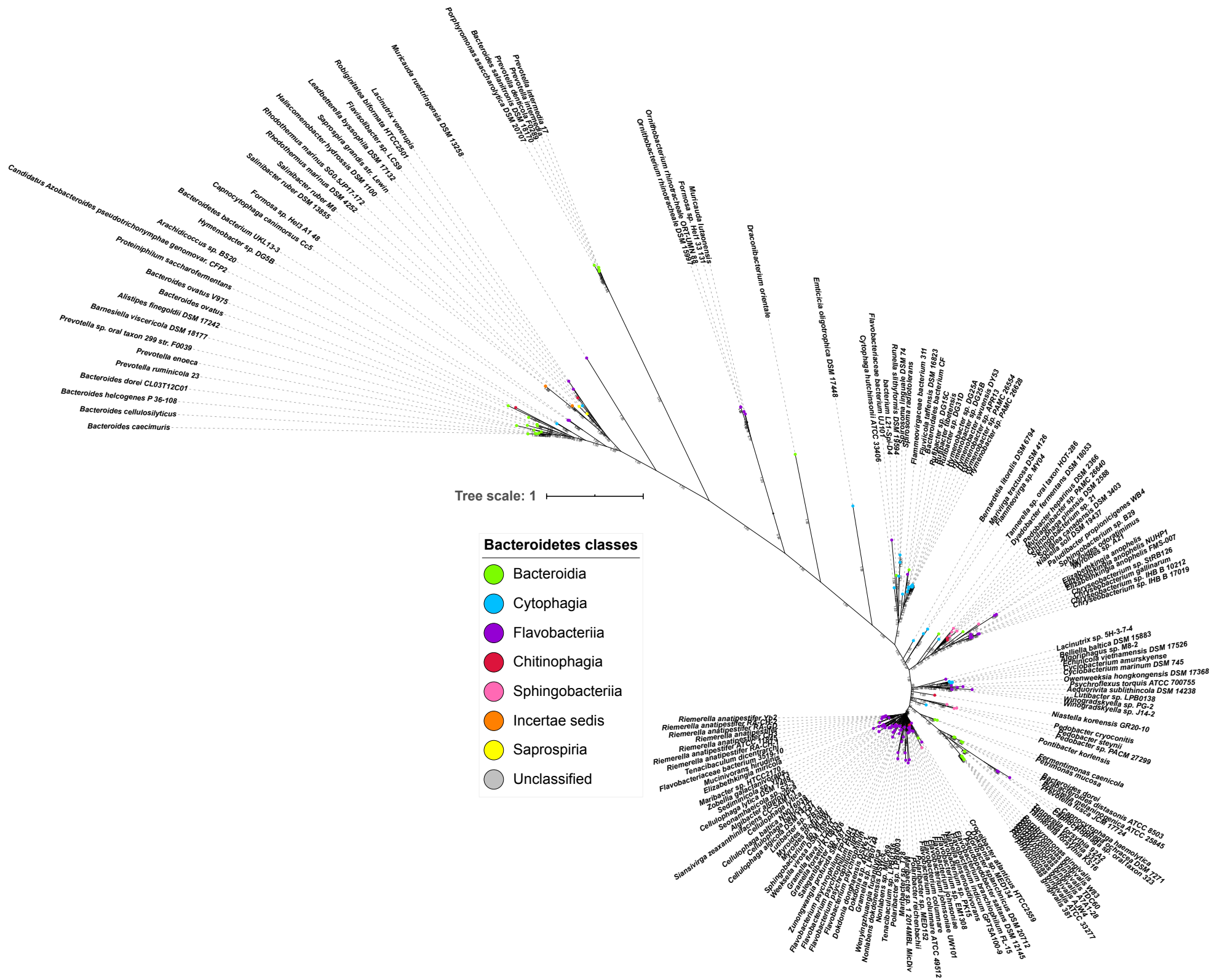

**Figure S22: The BI tree of UgdA with terminal nodes labelled with its corresponding species.**

Supplement: Supplemental Information 1 — T9SS components sequences, alignment of each T9SS protein family, NEX files used as input for MrBayes tool, genome annotation (GFF) file for Porphyromonas gingivalis and detailed Bayesian Inference trees with support values. [file peerj-08-9019-s001.zip › supplemental/Supplemental files/Supplemental Figure S22.pdf]
