# Supplementary material for: Phylogenetic comparison between Type IX Secretion System (T9SS) protein components suggests evidence of horizontal gene transfer
Source: PeerJ. 2020 Jun 26;8:e9019. doi: 10.7717/peerj.9019 (PMC7323717; doi:10.7717/peerj.9019)
Supplement: Supplemental Information 1 — T9SS components sequences, alignment of each T9SS protein family, NEX files used as input for MrBayes tool, genome annotation (GFF) file for Porphyromonas gingivalis and detailed Bayesian Inference trees with support values. [file Supplemental Figure S9.pdf › supplemental/Supplemental files/Supplemental Figure S9.pdf]

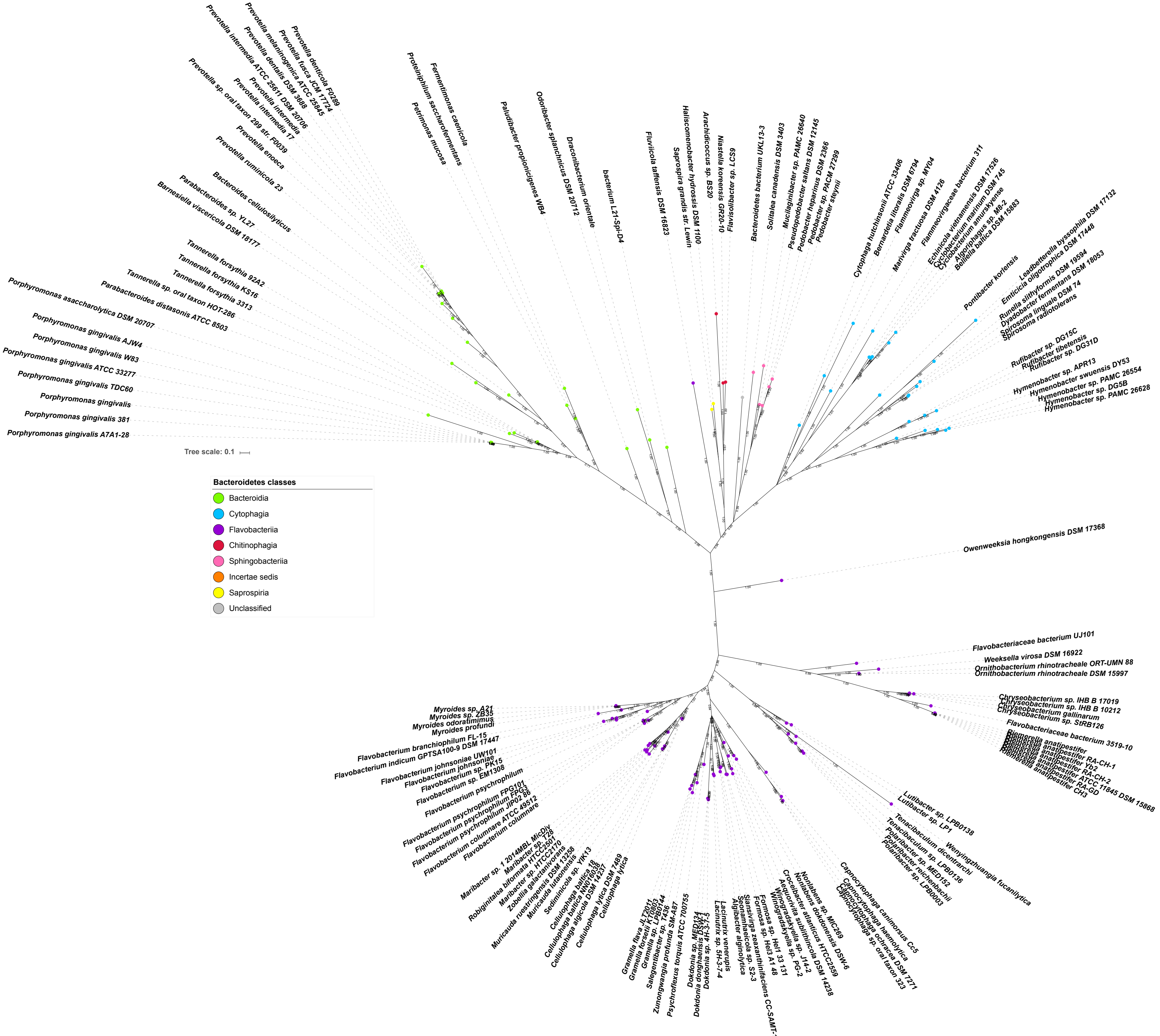

Figure S9: The BI tree of PorT with terminal nodes labelled with its corresponding species.
